# Prediction of return of spontaneous circulation during cardiopulmonary resuscitation by pulse-wave cerebral tissue oxygen saturation: a retrospective observational study

**DOI:** 10.1186/s12873-022-00586-9

**Published:** 2022-02-28

**Authors:** Kento Sakaguchi, Masayuki Takada, Kazunori Takahashi, Yu Onodera, Tadahiro Kobayashi, Kaneyuki Kawamae, Masaki Nakane

**Affiliations:** 1 Department of Emergency and Critical Care Medicine, Yamagata University Hospital, 2-2-2, Iida-nishi, Yamagata, Yamagata 990-9585 Japan; 2 Department of Anesthesiology, Yamagata University Hospital, 2-2-2, Iida-nishi, Yamagata, Yamagata 990-9585 Japan

**Keywords:** Cardiac arrest, Near-infrared spectroscopy, NIRO, SnO_2_, Return of spontaneous circulation

## Abstract

**Background:**

It is difficult to predict the return of spontaneous circulation (ROSC) during cardiopulmonary resuscitation (CPR). Cerebral tissue oxygen saturation during CPR, as measured by near-infrared spectroscopy (NIRS), is anticipated to predict ROSC. General markers of cerebral tissue oxygen saturation, such as the tissue oxygenation index (TOI), mainly reflect venous oxygenation, whereas pulse-wave cerebral tissue oxygen saturation (SnO_2_), which represents hemoglobin oxygenation in the pulse wave within the cerebral tissue, is an index of arterial and venous oxygenation. Thus, SnO_2_ may reflect arterial oxygenation to a greater degree than does TOI. Therefore, we conducted this study to verify our hypothesis that SnO_2_ measured during CPR can predict ROSC.

**Methods:**

Cardiac arrest patients who presented at the Emergency Department of Yamagata University Hospital in Japan were included in this retrospective, observational study. SnO_2_ and TOI were simultaneously measured at the patient’s forehead using an NIRS tissue oxygenation monitor (NIRO 200-NX; Hamamatsu Photonics, Japan). We recorded the initial, mean, and maximum values during CPR. We plotted receiver operating characteristic curves and calculated the area under the curve (AUC) to predict ROSC.

**Results:**

Forty-two patients were included. SnO_2_ was significantly greater in the ROSC group than in the non-ROSC group in terms of the initial (37.5% vs 24.2%, *p* = 0.015), mean (44.6% vs 10.8%, *p* < 0.001), and maximum (79.7% vs 58.4%, *p* < 0.001) values. Although the initial TOI was not significantly different between the two groups, the mean (45.1% vs 36.8%, *p* = 0.018) and maximum (71.0% vs 46.3%, *p* = 0.001) TOIs were greater in the ROSC group than in the non-ROSC group. The AUC was 0.822 for the mean SnO_2_ (95% confidence interval [CI]: 0.672–0.973; cut-off: 41.8%), 0.821 for the maximum SnO_2_ (95% CI: 0.682–0.960; cut-off: 70.8%), and 0.809 for the maximum TOI (95% CI: 0.667–0.951; cut-off: 49.3%).

**Conclusion:**

SnO_2_ values measured during CPR, including immediately after arrival at the emergency department, were higher in the ROSC group than in the non-ROSC group.

## Background

Cardiac arrest has a very poor prognosis and it is difficult to predict the return of spontaneous circulation (ROSC) during cardiopulmonary resuscitation (CPR). Cerebral tissue oxygen saturation represents the oxygenation of hemoglobin in all regional vessels within the cerebral tissue. It is easily measured in a non-invasive, real-time manner by near-infrared spectroscopy (NIRS) using probes attached to the patient’s forehead [1, 2]. It has been suggested that cerebral tissue oxygen saturation in patients with ROSC increases during CPR, and exceeds that in patients without ROSC [3–8]. In addition, cerebral tissue oxygen saturation may indicate the quality of CPR [3, 9]. Although physiological monitoring of CPR also includes end-tidal carbon dioxide (ETCO_2_) level and arterial blood pressure [10], the measurement of cerebral tissue oxygen saturation is quick and easy to perform. Therefore, cerebral tissue oxygen saturation during CPR may be a useful predictor of ROSC. However, valid criteria, including cut-off values for cerebral tissue oxygen saturation to predict ROSC, have not been determined [11, 12].

Pulse-wave cerebral tissue oxygen saturation (SnO_2_) is a marker of cerebral tissue oxygen saturation that represents the change in the oxygenated hemoglobin concentration as a percentage of the change in the total hemoglobin concentration [9]. Although general cerebral tissue oxygen saturation, including the tissue oxygenation index (TOI), represents the oxygenation of mainly stagnated hemoglobin in the regional vessels [13], SnO_2_ may reflect fluctuations in blood flow through all vessels in the regional tissue [9]. Because high quality CPR is important to achieve ROSC, SnO_2_ may be useful for predicting ROSC since it is influenced by the fluctuations in systemic blood flow caused by CPR. Furthermore, it has been suggested that SnO_2_ could be a useful indicator of the quality of CPR [9]. However, to the best of our knowledge, no study has investigated whether SnO_2_ predicts ROSC.

In this retrospective observational study, we investigated SnO_2_ values during CPR to verify our hypothesis that SnO_2_ during CPR predicts ROSC in patients arriving at an emergency department (ED).

## Methods

### Study design and setting

This was a retrospective, single-center, observational study. Patients aged > 18 years with out-of-hospital cardiac arrest were eligible if they arrived in a state of cardiac arrest at the ED at Yamagata University Hospital, a tertiary hospital in Japan, between October 2017 and September 2019. Only patients whose cerebral tissue oxygen saturation was recorded during CPR at the ED were eligible. All patients were treated in accordance with the Japan Resuscitation Council Guidelines 2015 [14]. The lead emergency physician decided when to terminate resuscitation. The medical staff did not alter the patient’s treatment based on the cerebral tissue oxygen saturation.

### Instrument

An NIRS tissue oxygenation monitor (NIRO 200-NX; Hamamatsu Photonics, Tokyo, Japan) was used in NIRO-Pulse mode for all patients. The measurement interval was set at 0.05 s. SnO_2_ and TOI were measured in all patients. SnO_2_ represents the change in the oxygenated hemoglobin concentration as a percentage of the change in the total hemoglobin concentration in all vessels within the tissue, including the arteries, capillaries, and veins. TOI represents the oxygenated hemoglobin concentration as a percentage of the total hemoglobin concentration in all vessels within the tissue region. If there are measurement errors, SnO_2_ may be recorded as a negative value, although it is unknown why this occurs.

### Measurements

NIRO probes were placed on the patient’s forehead on arrival at the ED. Cerebral tissue oxygen saturation was recorded in NIRO-Pulse mode during resuscitation. The initial SnO_2_ and TOI were each averaged over a 1-min period after the start of monitoring. The mean SnO_2_ and TOI were calculated as the mean values from the start of monitoring to the end of CPR. The maximum SnO_2_ and TOI were the maximum values recorded at any time during CPR.

### Statistical analyses

The primary outcomes of this study were the area under the curve (AUC) for SnO_2_ and TOI during CPR to predict ROSC. We plotted receiver operating characteristic curves to calculate the cut-off values. The secondary outcomes were the changes in cerebral tissue oxygenation saturation at 5 or 10 min after the start of measurement. We calculated the sample size required for the primary outcome of this study using the SnO_2_ data from the first 24 patients eligible for this study. With a type I error rate of 5% and a power of 80%, we determined that 36 patients were required overall.

Patient characteristics were compared using the Wilcoxon rank-sum test or *χ*^2^ test, as appropriate. SnO_2_ and TOI were compared between patients with ROSC (ROSC group) and patients without ROSC (non-ROSC group) using the Wilcoxon rank-sum test or Fisher’s exact test, as appropriate. The AUCs were compared using DeLong’s test. Statistical significance was set at *p* < 0.05. All statistical analyses were performed with Microsoft R Open version 3.5.3 (R Foundation for Statistical Computing, Vienna, Austria).

## Results

### Patient characteristics

Forty-two patients with a median age of 81.5 years (interquartile range: 68.3–87.5 years) were included in this analysis. Cardiac arrest was witnessed in 40.5% of patients. Bystander CPR was administered before the arrival of emergency medical services (EMS) in 73.8% of patients. ROSC was achieved in 31.0% of patients (Table 1). Three patients (7.1%) were defibrillated by a citizen or the EMS, 11 patients (26.2%) had arrest due to pulseless electrical activity (PEA), and 28 patients (66.7%) were in asystole when the EMS arrived. The major causes of cardiac arrest were acute coronary syndrome (21.4%), airway obstruction (14.3%), heart disease without acute coronary syndrome (9.5%), and renal failure (9.5%). Only one patient survived longer than 1 month after cardiac arrest.

**Table 1:** Baseline characteristics of the study population

Characteristics	Total	ROSC group	Non-ROSC group	*p* value
	***n* = 42**	***n* = 13 (31.0%)**	***n* = 29 (69.0%)**	
Age, median (IQR)	81.5 (68.3–87.5)	83 (75.0–91.0)	81 (68.0–86.0)	0.220
Sex, male, *n* (%)	26 (61.9)	5 (38.5)	21 (72.4)	0.079
Witnessed cardiac arrest, *n* (%)	17 (40.5)	7 (53.8)	10 (34.5)	0.399
Bystander CPR, *n* (%)	31 (73.8)	8 (61.5)	23 (79.3)	0.405
Initial rhythm on EMS arrival, *n* (%)				
Defibrillation	3 (7.1)	1 (7.7)	2 (6.9)	1.000
PEA	11 (26.2)	5 (38.5)	6 (20.7)	0.406
Asystole	28 (66.7)	7 (53.8)	21 (72.4)	0.409

*Abbreviations*: *ROSC* return of spontaneous circulation, *IQR* interquartile range, *CPR* cardiopulmonary resuscitation, *PEA* pulseless electrical activity

### Cerebral tissue oxygen saturation

**Fig. 1:**
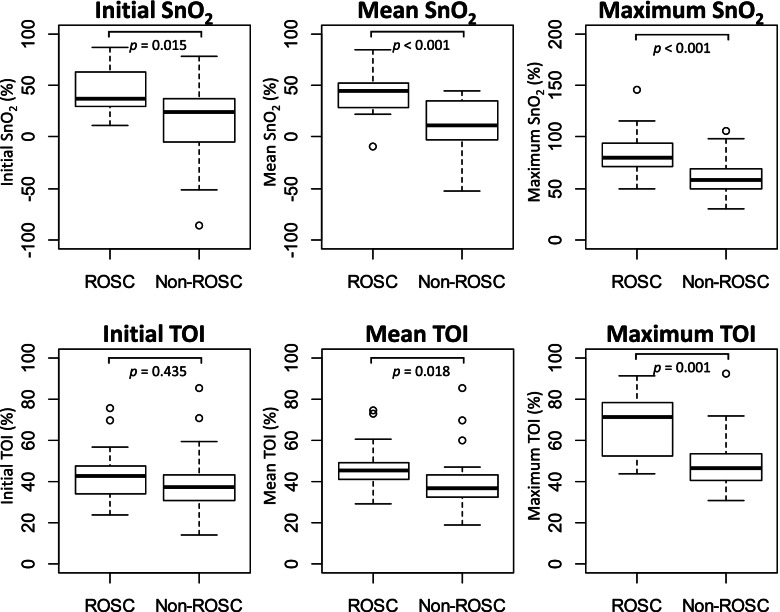
Box-and-whisker plots of cerebral tissue oxygen saturation data of the ROSC and non-ROSC groups

**Fig. 2:**
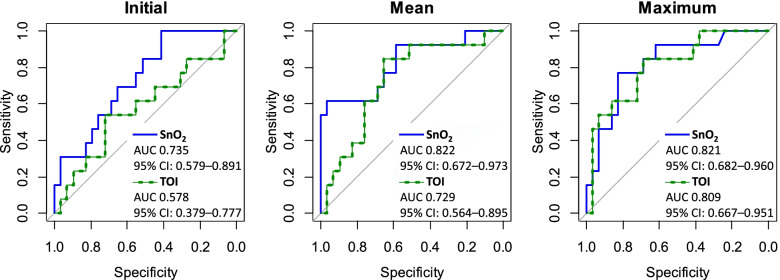
Receiver operating characteristic curves of SnO_2_ and TOI for predicting ROSC. Receiver operating characteristic curves were plotted with the initial, mean, and maximum values of SnO_2_ and TOI to describe ROSC and non-ROSC. Abbreviations: AUC, area under the curve; CI, confidence interval; ROSC, return of spontaneous circulation; SnO_2_, pulse-wave tissue oxygen saturation; TOI, total oxygenation index

The initial, mean, and maximum SnO_2_ were significantly greater in the ROSC group than in the non-ROSC group (Table 2, Fig. 1). Although the mean and maximum TOIs were greater in the ROSC group than in the non-ROSC group, the initial TOI was not significantly different between the two groups. The largest AUC was 0.822 for the mean SnO_2_, followed by 0.821 for the maximum SnO_2_, and 0.809 for the maximum TOI (Fig. 2). The AUCs for the initial, mean, and maximum values were not significantly different between SnO_2_ and TOI (Table 3).

**Table 2:** Differences in cerebral tissue oxygen saturation between the ROSC and non-ROSC groups

	**ROSC** **median (IQR)**	**Non-ROSC** **median (IQR)**	***p* value**
SnO_2_ (%)			
Initial	37.5 (29.1–62.8)	24.2 (− 4.8 to 37.0)	0.015
Mean	44.6 (28.1–51.9)	10.8 (− 2.8 to 35.5)	< 0.001
Maximum	79.7 (71.7–93.5)	58.4 (49.7–69.3)	< 0.001
TOI (%)			
Initial	42.7 (34.2–47.6)	37.2 (30.7–43.1)	0.435
Mean	45.1 (41.1–49.4)	36.8 (32.3–43.2)	0.018
Maximum	71.0 (52.2–78.1)	46.3 (40.4–53.4)	0.001

*Abbreviations*: *ROSC* return of spontaneous circulation, *IQR* interquartile range, *SnO*_*2*_ pulse-wave tissue oxygen saturation, *TOI* total oxygenation index

**Table 3:** Comparisons of the AUCs of SnO_2_ and TOI for ROSC

	**SnO**_**2**_ **AUC (95% CI)**	**TOI** **AUC (95% CI)**	***p* value**
Initial	0.735 (0.579–0.891)	0.578 (0.379–0.777)	0.091
Mean	0.822 (0.672–0.973)	0.729 (0.564–0.895)	0.273
Maximum	0.821 (0.682–0.960)	0.809 (0.667–0.951)	0.873

*Abbreviations*: *SnO*_*2*_ pulse-wave tissue oxygen saturation, *AUC* area under the curve, *CI* confidence interval, *TOI* total oxygenation index, *ROSC* return of spontaneous circulation

We also evaluated the increments in SnO_2_ and TOI at 5 or 10 min after arrival at the ED in the ROSC and non-ROSC groups. However, there were no statistically significant differences in these values (Table 4).

**Table 4:** Comparison of changes in cerebral tissue oxygen saturation at 5 or 10 min after admission to the ED between the ROSC and non-ROSC groups

	**ROSC** **median (IQR)**	**Non-ROSC** **median (IQR)**	***p* value**
Change in SnO_2_ at 5 min (%)	− 1.2 (− 11.9 to 10.3)	− 7.5 (− 23.4 to 7.0)	0.639
Change in SnO_2_ at 10 min (%)	− 8.5 (− 40.4 to 16.9)	− 11.8 (− 31.1 to 7.1)	0.901
Change in TOI at 5 min (%)	0.9 (0.3–3.2)	− 0.1 (− 2.0 to 1.3)	0.189
Change in TOI at 10 min (%)	1.7 (− 1.6 to 11.1)	− 0.1 (− 0.9 to 1.9)	0.329

*Abbreviations*: *ED* emergency department, *ROSC* return of spontaneous circulation, *IQR* interquartile range, *SnO*_*2*_ pulse-wave tissue oxygen saturation, *TOI* total oxygenation index

## Discussion

Cardiac arrest is a serious event and ROSC is not always possible, despite high-quality CPR. It is important to be able to predict whether a patient is likely to achieve ROSC during CPR. The ETCO_2_ level has been reported as a predictor of ROSC [15], but the measurement of ETCO_2_ required airway management with intubation or a supraglottic airway device.

Cerebral tissue oxygen saturation during CPR is greater in patients with ROSC than in those without [3–7, 11]. Furthermore, cerebral tissue oxygen saturation during CPR is correlated with cardiac output [16]. This may imply that the increase in cardiac output, as generated by high-quality CPR, may increase systemic organ perfusion, systemic oxygenation, pulmonary perfusion, and cerebral tissue oxygen saturation, leading to the achievement of ROSC combined with improved coronary perfusion and oxygenation [17]. Therefore, elevated cerebral tissue oxygen saturation during CPR may reflect an improvement in systemic organ perfusion.

**Fig. 3:**
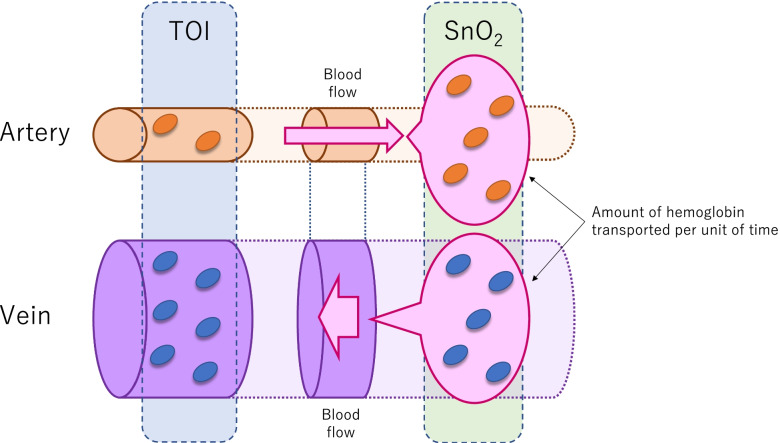
Difference between TOI and SnO_2_ measurements. Tissue oxygenation represents hemoglobin oxygenation in all regional vessels. TOI may predominantly reflect venous oxygenation. SnO_2_ may predominantly represent oxygenation in the hemoglobin transported in blood, and may reflect arterial oxygenation better than TOI. Abbreviations: TOI, total oxygenation index; SnO_2_, pulse-wave tissue oxygen saturation

In this study, we evaluated TOI and SnO_2_, two parameters measured by NIRS, as markers of cerebral tissue oxygen saturation. TOI is commonly used as a marker of regional hemoglobin oxygenation. Because hemoglobin is distributed throughout all vessels, including the arteries, capillaries, and veins, it reflects hemoglobin oxygenation in all blood vessels within the tissue region. TOI primarily represents venous oxygenation because the venous blood volume is greater than the arterial volume in regional tissues [13]. In comparison, SnO_2_ is calculated as the change in the oxygenated hemoglobin concentration as a percentage of the change in the total hemoglobin concentration. Accordingly, it may represent the blood flow in all regional vessels that is generated by chest compression. Because the amount of hemoglobin moved by blood flow is approximately equal in arteries and veins, it is hypothesized that SnO_2_ is a better marker of arterial oxygenation than is TOI, which mainly represents venous oxygenation (Fig. 3). Consequently, SnO_2_ has been proposed as an indicator of the quality of CPR [9]. However, few studies have examined the utility of SnO_2_ during CPR.

In this study, SnO_2_ and TOI were significantly greater in the ROSC group than in the non-ROSC group, except for the initial TOI. It has been reported that cerebral tissue oxygen saturation during CPR after arrival at the ED was greater in a ROSC group than in a non-ROSC group [3–7, 11], but it has not been confirmed whether cerebral tissue oxygen saturation at the time of arrival at the ED is already greater in cardiac arrest patients with ROSC than in those without. Cerebral tissue oxygen saturation may be lower prior to arrival at the ED than at the time of arrival [18]. This may be due to the etiology of cardiac arrest, as well as pre-hospital factors, such as the interval from cardiac arrest to the start of CPR and the quality of CPR administered by bystanders or the EMS. Such factors may be important predictors of ROSC [18]. Interestingly, we found that the initial SnO_2_ was greater in the ROSC group at the time of arrival. This may indicate that the systemic oxygenation during CPR in patients in the ROSC group may already be improving at the time of arrival at the ED.

The AUCs for predicting ROSC were greatest for the mean SnO_2_, maximum SnO_2_, and maximum TOI. Therefore, these findings suggest that the mean and maximum SnO_2_, and the maximum TOI may be predictors of ROSC during CPR. However, the increases in cerebral tissue oxygen saturation at 5 or 10 min after arrival at the ED were not significantly different between the ROSC and non-ROSC groups. Several studies have reported that the maximum cerebral tissue oxygen saturation or the magnitude of the increase in oxygen saturation during CPR may be useful predictors of ROSC [5, 8]. However, the mean values, the maximum values, and the magnitudes of the increases during CPR are not immediately available to physicians in the ED. In contrast, an advantage of measuring the initial SnO_2_ is that it can be measured quickly, within about 1 min, upon arrival at the ED.

SnO_2_, which reflects arterial and venous oxygenation, may provide a more useful assessment of arterial oxygenation than TOI, which mainly reflects venous oxygenation. Thus, the finding that the initial SnO_2_ is higher and the initial TOI is similar in ROSC patients compared with non-ROSC patients may indicate that arterial oxygenation is already starting to improve when ROSC patients arrive at the ED, but that venous oxygenation has not yet improved. Additionally, the observation that the mean TOI is higher in ROSC patients than in non-ROSC patients, although the increments in TOI after 5 and 10 min are similar between the two groups, suggests that TOI may increase just prior to ROSC [9]. Consequently, venous oxygenation may only show signs of improvement once ROSC is achieved. These results suggest a mechanism underlying the improvement in systemic oxygenation during CPR in which arterial oxygenation in ROSC patients may already be improving by the time of arrival at the ED, and venous oxygenation may improve once sufficient oxygen has been supplied to the cerebral tissue by CPR. Further studies are needed to verify this mechanism.

Cerebral tissue oxygen saturation can be measured quickly by placing an NIRS probe on the forehead. Although we could not conclude that SnO_2_ is an independent predictor of ROSC because of the univariate analysis, our study has revealed that, unlike TOI, the initial SnO_2_ values measured at admission to the ED were already high during CPR in ROSC patients. Based on these findings, we believe that SnO_2_ may become an indicator for predicting ROSC and will help advance resuscitation medicine.

### Limitations

The limitations of this study are as follows. First, although the sample size was calculated appropriately to assess the primary outcome, a larger number of patients is necessary to determine the associations of SnO_2_ and TOI with the overall prognosis and neurological outcomes. The patients in this study had poor prognoses because the initial rhythm was PEA or asystole upon the arrival of EMS. Second, it was difficult to maintain the accuracy of the measurements because slight deviations in the attachment of the probe can introduce measurement error. It is difficult to assess measurement error and we could not exclude this error. Third, because this was a retrospective study, we were unable to investigate the influence of some factors, such as pre-hospital and in-hospital CPR hours, which were not available in medical records. Fourth, this study does not examine the factors assessed in this study, such as SnO_2_, can be used as indicators of the quality of CPR. Fifth, it may be hypothesized that chest compression may cause venous retrograde flow and arterial antegrade flow to the brain, although this hypothesis has not been proven and the effect of this phenomenon on SnO_2_ during CPR remains uncertain.

## Conclusion

SnO_2_ values measured during CPR, including immediately after arrival at the ED, were higher in the ROSC group than in the non-ROSC group. We also found that in patients who achieved ROSC, arterial oxygenation may already be showing signs of improvement by the time of arrival at the ED.

## Data Availability

The datasets used during the current study are available from the corresponding author on reasonable request.

## Abbreviations

ROSC: Return of spontaneous circulation
SnO_2_: Pulse-wave tissue oxygen saturation
TOI: Total oxygenation index
